# Addressing the impact of urban exposure on the incidence of type 2 diabetes mellitus: The PERU MIGRANT Study

**DOI:** 10.1038/s41598-018-23812-6

**Published:** 2018-04-03

**Authors:** Andrea Ruiz-Alejos, Rodrigo M. Carrillo-Larco, J. Jaime Miranda, Cheryl A. M. Anderson, Robert H. Gilman, Liam Smeeth, Antonio Bernabé-Ortiz

**Affiliations:** 1 CRONICAS Center of Excellence in Chronic Diseases, Universidad Peruana Cayetano Heredia, Lima, Peru; 2 Department of Medicine, School of Medicine, Universidad Peruana Cayetano Heredia, Lima, Peru; 3 Department of Family Medicine and Public Health, School of Medicine, University of California San Diego. La Jolla, California, USA; 4 Department of International Health, Bloomberg School of Public Health, Johns Hopkins University, Baltimore, USA; 5 Faculty of Epidemiology and Population Health, London School of Hygiene and Tropical Medicine, London, United Kingdom

## Abstract

The aim of this study was to estimate the incidence of T2DM in three population groups: rural, rural-to-urban migrants and urban dwellers. Data from the PERU MIGRANT Study was analysed. The baseline assessment was conducted in 2007–2008 using a single-stage random sample and further follow-up was undertaken in 2015–16. T2DM was defined based on fasting glucose and self-reported diagnosis. Poisson regression models and robust variance to account for cluster effects were used for reporting risk ratios (RR) and 95%CI. At baseline, T2DM prevalence was 8% in urban, 3.6% in rural-to-urban migrants and 1.5% in rural dwellers. After 7.7 (SD: 1.1) years, 6,076 person-years of follow-up, 61 new cases were identified. The incidence rates in the urban, migrant and rural groups were 1.6, 0.9 and 0.5 per 100 person-years, respectively. Relative to rural dwellers, a 4.3-fold higher risk (95%CI: 1.6–11.9) for developing T2DM was found in urban dwellers and 2.7-fold higher (95%CI: 1.1–6.8) in migrants with ≥30 years of urban exposure. Migration and urban exposure were found as significant risk factors for developing T2DM. Within-country migration is a sociodemographic phenomenon occurring worldwide; thus, it is necessary to disentangle the effect of urban exposure on non-healthy habits and T2DM development.

## Introduction

Type 2 diabetes mellitus (T2DM), a major non-communicable disease (NCD)^1^, accounted for 1.5 million deaths worldwide in 2013. Most type 2 diabetes occurs in low-and-middle income countries (LMICs)^2^. Globally, the burden of type 2 diabetes has increased: over 30 years, its prevalence has almost doubled in men (4% to 9%) and women (5% to 8%)^3^. Specifically, in the Americas, T2DM prevalence in 2014 was estimated to be around 8.5%^4^, whereas this estimate reached 7% in Peru in 2012 and an estimated incidence of 1.95 per 100 person-years^5,6^.

Globalization, urbanization, migration and upward social mobility have been associated with pro-diabetic and obesogenic lifestyle behaviors, especially among poorly educated migrants or migrants of low socioeconomic status^7–9^. The phenomenon called “nutritional transition” has been pivotal in understanding the increased risks of overnutrition and related conditions such as overweight/obesity and T2DM^9^. Among within-country migrants, the nutritional transition signals the environmental change that individuals face following rural-to-urban migration, thus potentially explaining the elevated risk of developing some chronic cardiometabolic conditions^9–11^. Rural-to-urban migrants, compared to those who remain in their rural region, tend to acquire sedentary behaviors and to have “less healthy” nutritional habits^12–15^.

The effect of urban exposure is associated with different factors, some of which are inherent to particular population groups. From a biological and mechanistic point of view, the thrifty phenotype hypothesis suggests that T2DM and obesity risk is greater in those exposed to fetal under-nutrition, a trait that is more pronounced in deprived sectors of society, including rural sites. The adversity *in utero* may lead to an increase in insulin resistance among other metabolic changes, beneficial for survival during the intrauterine period. Yet, the same adaptive process could potentially affect cardiovascular health later in life, and therefore is likely to affect the health of rural dwellers and rural-to-urban migrants in adulthood^16^. This hypothesis has been tested in studies in India and China and highlights the influence of environmental exposure, i.e. urbanization and migration, especially for those with early deprivation, in the risk for developing T2DM^17,18^.

Most studies assessing the impact of migration on lifestyles and cardiovascular risks have primarily focused on international migration^19,20^. However, longitudinal studies approximating the incidence of the main cardiovascular risk factors among within-country migrants are scarce. Usually, selection bias might arise in these studies as a great percentage of international migrants could either be healthier or have higher socioeconomic status allowing them more resources to cope with their receiving host environments, primarily urban settings in developed societies^20^. Additionally, literature regarding the impact of rural-to-urban within-country migration on health is limited to cross-sectional or retrospective reports instead of prospective studies^12^. As within-country migration is a global phenomenon occurring swiftly, both in time and in volume, in LMICs, population-based cohort surveys are needed to understand how rural-to-urban migration and urban exposure play a role in the development of T2DM.

Between the 1960' s and 1990′ s, the internal migration patterns in Peru increased due to political violence^21^, thus becoming one of the major drivers for migration from rural to urban areas and reducing, at the same time, selection biases intrinsically related to migration processes^22^. Initially, it is likely that the first migrants were those with higher socioeconomic status, but as migration rates increased during 1970–1980 s, it occurred regardless of the migrants’ socioeconomic status. The PERU MIGRANT Study was established to determine cross-sectional and longitudinal differences in cardiometabolic risk factors between rural, rural-to-urban migrant and urban populations^23^. We hypothesized that the exposure to an urban environment would increase the risk associated with developing T2DM.

## Results

### Characteristics of the study population at baseline

A total of 988 individuals were enrolled at baseline; 20.3% rural, 59.6% migrant (72.1% migrated when ≥14 years old), and 20.1% urban. Of the total sample, 524 (53%) were females, and the overall mean age was 48 years (SD: 12.0). The overall prevalence of T2DM at baseline was 4.1% (95% CI 2.8–5.3%). T2DM prevalence was higher in the urban group compared to the migrants and the rural dwellers, 8%, 3.6%, and 1.5%, respectively (p-value = 0.003). In addition, older age, obesity, metabolic syndrome, and hypercholesterolemia were associated with T2DM status at baseline (E-Table 1).

### Follow-up and incidence of T2DM

**Figure 1:**
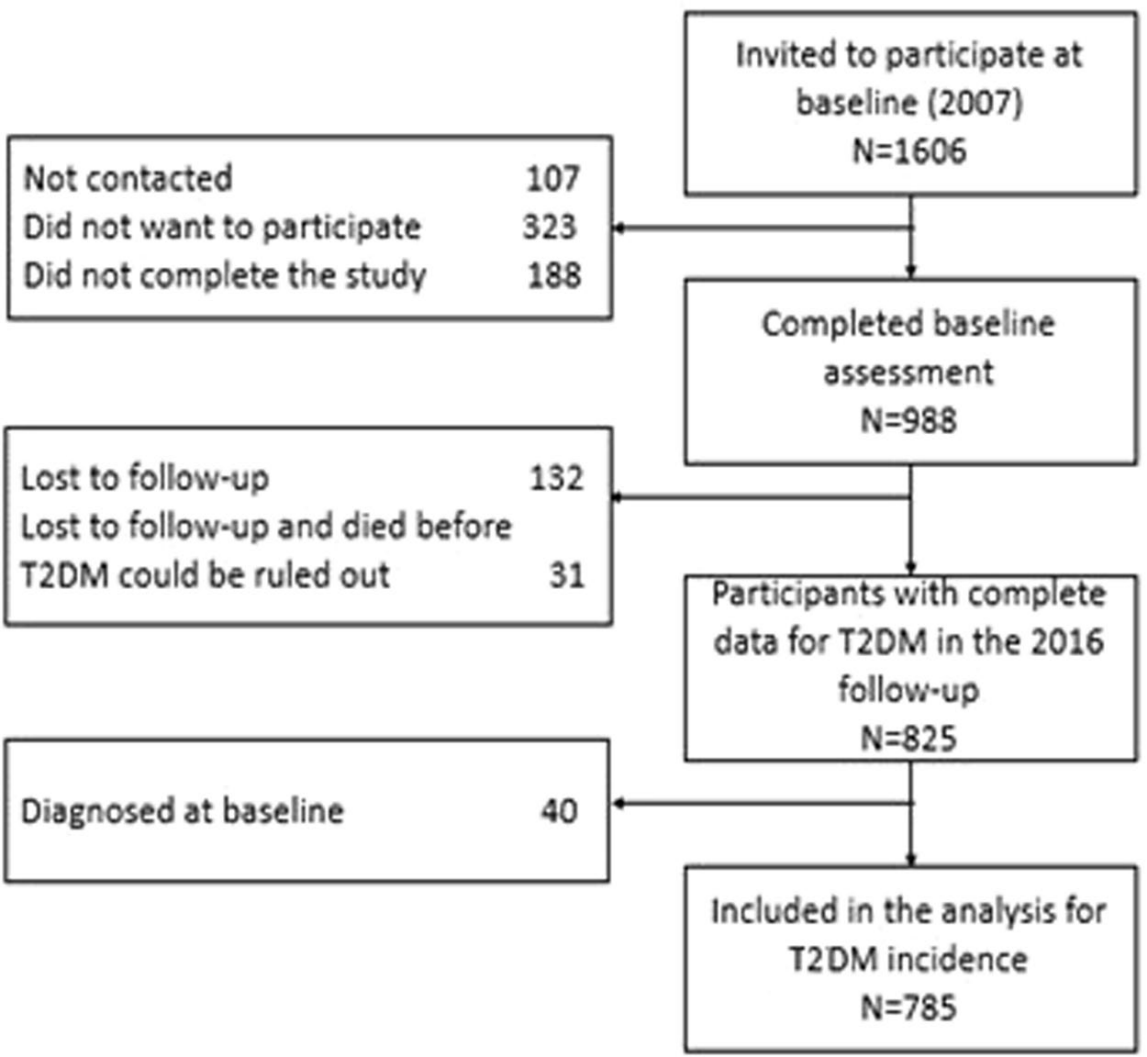
Baseline enrolment and participants included from the second follow-up of the PERU MIGRANT study.

A total of 163 (16.5%) participants were lost to follow-up (27%, 13.6%, and 14.6% in rural, rural-to-urban migrant and urban groups, respectively), and 57 (5.8%) died prior to follow-up. In addition, we excluded those individuals with T2DM at baseline (n = 40). Thus, data of 785 participants were included in the incidence analysis (Fig. 1).

The mean follow-up time was 7.7 years (SD: 1.1), resulting in 6,076 person-years. A total of 61 new cases of T2DM were identified, corresponding to an overall incidence of 1.0 (95% CI 0.8–1.3) per 100 person-years. According to the population groups, cumulative incidence was 1.6 (95% CI 1.0–2.6) in the urban, 1.0 (95% CI 0.7–1.3) in the rural-to-urban migrants, and 0.6 (95% CI 0.3–1.2) in the rural group (Table 1). In the univariate model, those who were male, obese and had metabolic syndrome at baseline showed evidence of having a higher risk of developing T2DM (Table 1). Of note, neither socioeconomic status nor non-healthy lifestyle variables were associated with the outcome of interest.

**Table 1:** Incidence and risk ratio of Type 2 Diabetes according to population characteristics and risk factors.

	Incidence (95% CI) per 100 person-years	Risk ratio (95% CI) Univariate model	p-value
***Sex***			
Female	1.23 (0.90–1.67)	1 (Reference)	
Male	0.71 (0.47–1.13)	0.60 (0.35–1.03)	0.06
***Age***			
<50 years	0.88 (0.62–1.26)	1 (Reference)	
50+ years	1.17 (0.82–1.67)	1.43 (0.86–2.36)	0.17
***Asset index***			
Low	1.09 (0.75–1.58)	1 (Reference)	
Middle	1.08 (0.66–1.77)	0.94 (0.51–1.74)	0.85
High	0.84 (0.52–1.35)	0.74 (0.41–1.35)	0.32
***Education***			
None/Some	1.05 (0.68–1.63)	1 (Reference)	
Primary complete	0.84 (0.42–1.68)	0.75 (0.33–1.71)	0.50
Some secondary or more	1.03 (0.73–1.45)	0.89 (0.51–1.55)	0.51
***Current daily smoking***			
No	1.02 (0.79–1.31)	1 (Reference)	
Yes	0.50 (0.07–3.57)	0.46 (0.06–3.36)	0.45
***Heavy alcohol drinking***			
No	1.02 (0.79–1.32)	1 (Reference)	
Yes	0.79 (0.30–2.10)	0.76 (0.27–2.09)	0.59
***Physical activity levels***			
Moderate/High	1.06 (080–1.41)	1 (Reference)	
Low	0.89 (0.52–1.48)	0.81 (0.45–1.48)	0.50
***Obesity***			
No	0.65 (0.46–0.92)	1 (Reference)	
Yes	2.52 (1.75–3.62)	**3.90** (**2.36–6.43)**	**<0.001**
***Metabolic syndrome***			
No	0.53 (0.35–0.80)	1 (Reference)	
Yes	2.20 (1.60–3.02)	**4.19** (**2.50–7.03)**	**<0.001**
***Hypertension***			
No	0.95 (0.72–1.25)	1 (Reference)	
Yes	1.34 (0.76–2.36)	1.55 (0.82–2.91)	0.17
***Hypercholesterolemia***			
No	0.97 (0.71–1.32)	1 (Reference)	
Yes	1.07 (0.70–1.65)	1.13 (0.67–1.92)	0.64

### Urban exposure and the risk of developing type 2 diabetes

After adjusting for confounding factors (Table 2), urban dwellers (RR = 4.34; 95% CI: 1.58–11.92) were at higher risk of developing T2DM when compared to rural population (Model 1); however, this association was not evident after controlling for obesity (RR = 2.36; 95% CI: 0.83–6.72; Model 2). On the other hand, migrant group did not evidence significant risk of developing T2DM in either Model 1 (RR = 2.21; 95% CI: 0.95–5.69) nor Model 2 (RR = 1.59; 95% CI: 0.64–6.72).

**Table 2:** Risk of developing Type 2 Diabetes by population group: Crude and adjusted models.

	Incidence (95% CI)	Crude model	Adjusted model 1*	Adjusted model 2**
	per 100 person-years	RR (95% CI)	RR (95% CI)	RR (95% CI)
	(n = 714)	(n = 714)	(n = 707)	(n = 707)
*Population group*				
Rural	0.55 (0.25–1.21)	1 (Reference)	1 (Reference)	1 (Reference)
Migrant	0.95 (0.68–1.31)	1.74 (0.73–4.13)	2.32 (0.95–5.69)	1.59 (0.64–3.93)
Urban	1.63 (1.04–2.55)	**2.86** (**1.14–7.16)**	**4.34** (**1.58–11.92)**	2.36 (0.83–6.72)

In bold: p < 0.05.

*Model adjusted by age, sex, education and assets index, current daily smoking, heavy alcohol drinking, and physical activity levels. Using an alternative categorization based on four age categories (<40, 40–49, 50–59, 60+) gives similar results to those based on two age categories.

**Model adjusted by age, sex, education, assets index, current daily smoking, heavy alcohol drinking, physical activity levels and obesity. Using an alternative categorization based on four age categories (<40, 40–49, 50–59, 60+) gives similar results to those based on two age categories.

When years lived in urban area was used as the exposure instead of population group, and demographic confounders and behavioural risk factors were controlled for, compared to rural dwellers, those migrants living for 30+ years in urban settings were at higher risk (RR = 2.70; 95% CI: 1.17–6.83) of developing T2DM (Table 3, Model 1). Of note, this effect disappeared after controlling for obesity (Table 3, Model 2). Age at migration, however, was not associated with developing T2DM in any of the models.

**Table 3:** Risk of developing Type 2 Diabetes by acculturation surrogates: Crude and adjusted models.

	Crude	Adjusted model 1*	Adjusted model 2**
	RR (95% CI)	RR (95% CI)	RR (95% CI)
***Years lived in urban area***	(n = 693)	(n = 686)	(n = 686)
Rural (never)	1 (Reference)	1 (Reference)	1 (Reference)
Migrant of <30 years	0.96 (0.34–2.70)	1.28 (0.41–4.01)	0.93 (0.31–2.77)
Migrant of ≥30 years	2.21 (0.90–5.43)	**2.70 (1.17–6.83)**	1.94 (0.75–5.02)
Urban (always)	**2.86 (1.14–7.16)**	**3.88 (1.38–10.90)**	2.33 (0.81–6.68)
***Age at first migration*** ^***†***^	(n = 437)	(n = 432)	(n = 432)
<14 years	1 (Reference)	1 (Reference)	1 (Reference)
≥14 years	1.54 (0.79–3.02)	1.47 (0.71–3.07)	1.23 (0.59–2.56)

In bold: p < 0.05.

*Model adjusted by age, sex, education and assets index, current daily smoking, heavy alcohol drinking, and physical activity levels. Using an alternative categorization based on four age categories (<40, 40–49, 50–59, 60+) gives similar results to those based on two age categories.

**Model adjusted by age, sex, education, assets index, current daily smoking, heavy alcohol drinking, physical activity levels and obesity. Using an alternative categorization based on four age categories (<40, 40–49, 50–59, 60+) gives similar results to those based on two age categories.

^†^Variable only assessed among rural-to-urban migrants.

## Discussion

Compared to the rural group, urban dwellers had a 4-fold greater risk of developing type 2 diabetes. Additionally, number of years lived in urban area was associated with increased risk of T2DM development but age at first migration was not, both of which are well-known surrogates of acculturation: migrants living ≥30 years in the urban environment had 2.7 higher risk of developing T2DM compared to the rural dwellers. Taken together, our findings demonstrate that the exposure to an urban environment, both a lifetime urban exposure and long term urban exposures following migration, is a key issue for the individual’s risk for T2DM.

To the best of our knowledge, this is one of the pioneer ongoing prospective studies directly assessing the impact of urban exposure on the progression towards T2DM in LMIC settings. Previous cross-sectional studies have reported the impact of international migration on cardiovascular risk factors, including T2DM^24,25^, finding that acculturation, assessed as length of residence, seemed to play an important role in the increased prevalence of T2DM. For example a cross-sectional study of an ethnically diverse sample of US immigrants found that those living in the US ≥10 years had a higher probability of being obese, having hypertension and T2DM compared to those residing <10 years^26^.

When within-country migration is evaluated, data regarding risk factors associated with T2DM incidence is more limited. Jaffe *et al*. analyzed data from a historic retrospective cohort analysis in order to assess the combined effect of migration and ethnicity of T2DM risk among Ethiopian and non-Ethiopian Jews^27^. However, a sub-analysis found an important risk feature for type 2 diabetes, more related to ethnicity than to migration: those born in Ethiopia had a greater probability of developing T2DM compared to those born in Israel. Other cross-sectional studies, mainly conducted in Asia, have reported an increased prevalence of T2DM in urban dwellers compared to rural-to-urban migrants and rural dwellers^24,25,28,29^. For example, in a cross-sectional study conducted in India, Ebrahim *et al*. reported that migration from rural to urban areas was associated with obesity and type 2 diabetes. Prevalence of T2DM was similar among urban dwellers and rural-to-urban migrants and higher than rural participants, both in women and men^29^. Similar findings showing a clear difference in T2DM prevalence between urban and rural populations were also reported in Iran^28^. Data of the PERU MIGRANT Study at baseline also confirmed this trend and our current study expands on previous reports by assessing an almost 8-year incidence of T2DM in these three populations.

According to the literature, urbanization has been assessed using different methods. For example, whereas some studies have utilized place of residence (i.e. urban vs. rural), others have used acculturation surrogates such as length of residence or age at first migration. Our study not only used the same surrogates but also characterized three well-defined population groups according to their migration exposures. For example, in another study conducted in Peru, the CRONICAS Cohort Study, our group did not find a clear difference in T2DM incidence between rural, semi-urban and urban settings, but did find a higher incidence risk among those living in high altitude settings^30^. Whilst the strength of the CRONICAS Cohort Study relies on current geographical expositions, the PERU MIGRANT Study utilized well defined population groups according to migrant and non-migrant profiles. Of note, one of the main results of the 5-year follow-up of the PERU MIGRANT Study was the increased risk of general and central obesity reported among the rural-to-urban migrant group and urban dwellers as compared to the rural population^31^. As obesity has been reported as the leading risk factor associated with T2DM^30^, our findings seem to be the consequence of exposure to urban areas, first leading to obesity and then progressing towards to type 2 diabetes.

When assessing incidence rates, our overall results are close to half of estimates previously published in Peru^6,30^. We believe these findings are because the PERUDIAB Study did not include rural settings^6^, or most of the sample was for urban and semiurban settings as in the CRONICAS Cohort Study^30^. Our study, however, included rural and rural-to-urban migrant populations, expected to have lower T2DM incidence.

Urbanization and rural-to-urban migration may lead to lifestyle changes that could have an impact on the development of NCDs worldwide. It has been described that urbanization leads to an obesogenic environment as many urban areas do not support healthy lifestyle choices^32^. Moreover, the risk of developing T2DM can also be increased if the thrifty phenotype is more prevalent in resource-constrained settings^32^. A relatively recent systematic review found that, in the United States, urban sprawl and less mixed land use were consistently associated with overweight and obesity among adults^33^. Although in this review, results show great heterogeneity regarding other physical environmental factors, it is expected that these factors have an impact on physical activity levels too. In addition, within-country migration and urbanization are two sociodemographic phenomena commonly occurring in developing countries undergoing rapid socioeconomic development. We believe our manuscript provides supporting evidence that urbanization is contributing to the risk of developing T2DM, particularly in limited-resource settings.

The main strength of this study relies on its well-defined population groups. In addition, as political violence was one of the main reasons for migration, this could attenuate bias due to self-selected migration. However, our study is not exempt from the healthy migrant effect^34^, observed in migrants with better socioeconomic and education status. Thus, our estimated risk ratios might have been biased downwards. On the other hand, Pampas de San Juan de Miraflores, where urban and migrant individuals were enrolled, was a very deprived area (households built with mud and thatch, and there were no electricity, water or sanitation services) almost comparable with rural areas in 1970 s. Thus, it is probably that only poor migrants and not those with better socioeconomic position settled there, and for instance our results could be biased upwards. Therefore, further studies will be needed to disentangle the effect of socioeconomic status, education and migration on T2DM.

Limitations of the study should be highlighted. First, only one fasting glucose assessment was used at baseline and follow-up, instead of using the oral glucose tolerance test, the recommended gold standard. This could increase the risk of misclassification (i.e. some T2DM cases may be considered as healthy individuals due to low sensitivity of the fasting glucose). However, since the same technique was used for the two assessments, any misclassification would change results to the null. Second, because we did not have the exact date when our participants developed T2DM, we followed actuarial methods used in epidemiological surveys and assumed that individuals developed the disease at the mid-point in time between the first and last follow-up evaluations. This information was used to estimate the incidence rates. Our results should, nonetheless, be interpreted cautiously as the rate of developing T2DM has been assumed to be constant over the follow-up period. Third, our results are not exempt from potential selection bias, as both baseline response rates and attrition rates were higher in rural dwellers, yet we were unable to ascertain if these losses are differential or not with regards to the outcome of interest. Fourth, the small sample size and attrition rates may also explain why some acculturation surrogates, e.g. age at first migration, did not have sufficient power to be extensively explored in the analysis. In addition, small sample size may explain why significance reduces after controlling for obesity markers. However, as we mentioned in the methods section, obesity is mostly considered an intermediate variable instead of a potential confounder. For instance, data from this cohort has previously demonstrated the association between longer length of urban exposure and higher risk of obesity^35^. Additionally, other variables for which adjustment has been made in our analyses such as physical activity or education may also be intermediate variables. Thus, more studies are needed to further disentangle the effects of urban exposure in developing type 2 diabetes. Finally, detailed diet patterns were not collected at baseline or at follow-up assessments. As diet patterns are one of the main components of the nutrition transition, future studies may include diet evaluations which could provide significant information for the development of T2DM.

Our study demonstrates a clear link between urban exposure and the risk of developing T2DM over the life course of migrant and non-migrant populations. Compared to rural dwellers, rural-to-urban migrants, particularly those exposed for ≥30 years to an urban environment, and urban participants had higher risks of developing type 2 diabetes. Appropriate strategies are required to reduce the risk of developing T2DM among migrant and urban dwellers in LMIC settings.

## Materials and Methods

### Study design

Data from the PERU MIGRANT Study^23^, a prospective ongoing cohort study, was analyzed. The baseline assessment was conducted in 2007–2008 and follow-up evaluations were carried out in 2012–13 and 2015–2016^36^. Data from the baseline assessment and the 2015–2016 follow-up are included in this analysis.

### Settings and Participants

The district of San Jose de Secce, in the province of Huanta (Ayacucho) was selected as the rural site. In Lima, Las Pampas de San Juan de Miraflores was selected as the urban environment. Individuals who were ≥30 years of age and permanently living in their place of residence were invited to participate at baseline. Pregnant women or potential participants unable to understand procedures and consent were excluded. Rural participants were enrolled in San Jose de Secce, while urban dwellers and rural-to-urban migrants were recruited from Las Pampas de San Juan de Miraflores in Lima^23^.

A single-stage random sampling technique was used for enrolment. The participants were randomly selected through census in their area of residence. Consequently, to be considered in the rural group, a participant must have been permanently living in San Jose de Secce. In Lima, using data from an updated local census made in 2007, individuals who reported having been born in Lima and permanently living in Las Pampas de San Juan de Miraflores were considered in the urban group; and individuals who reported having been born in Ayacucho but permanently living in Las Pampas de San Juan de Miraflores at the time of the study enrolment were considered in the migrant group^23^. For the follow-up, the participants were re-assessed by contacting them in the same settings where they were originally enrolled at baseline^36^.

### Definition of Variables

#### Outcome variable

The outcome of interest was type 2 diabetes. At baseline, T2DM diagnosis was considered if the patient met all of the following criteria: a fasting glucose level ≥126 mg/dL (≥7 mmol/L) obtained within 8 and 12 hours of fasting, self-report of T2DM diagnosis by a physician and self-report of receiving anti diabetic medication. In the 2015–2016 follow-up, new T2DM cases were assessed by a participant meeting at least one of the first two diagnostic criteria (i.e. follow-up assessment only included fasting blood sampling or self-report of T2DM diagnosis). Exact date of T2DM onset could not be obtained as clinical records were not available. For plasma glucose, we used an enzymatic colorimetric method (GOD-PAP, Modular P-E/Roche-Cobas, Grenzach-Whylen, Germany) in 5 ml of whole blood.

#### Exposure variables

The primary exposure of interest was the population group (rural, rural-to-urban migrant and urban) as defined at baseline. Additionally, we assessed the urban exposure using two different approaches: years lived in urban area and age at first migration, split into categories according to the median of the distribution. Years lived in urban area was defined as the self-reported time living in the urban place and then split into four groups: rural (i.e. never lived in an urban area), migrants with <30 years living in an urban area, migrants with ≥30 years living in an urban environment, and urban dwellers (i.e. those who reported living always in the urban area). Age at first migration from rural-to-urban area, was defined by the self-report of the age at which the participant left the village they were born in. This variable was only calculated for migrant population and then split into two groups: <14 and ≥14 years old at the time of rural out migration.

#### Other variables

Other variables assessed at baseline and included in the analysis as potential confounders were: sex (female vs. male); age (<50 vs. ≥50 years); education (none/some, primary complete and secondary or more); and socioeconomic status based on a wealth index and split in tertiles. The wealth index was based on the possession of household resources (radio, types of television, refrigerator, computer, telephone, cell phone, internet, cable TV, motorcycle, car and gas cooker). The following lifestyle risk factors were also considered: alcohol consumption, defined by heavy alcohol drinking based on reporting a hangover or ≥6 drinks on the same occasion at least once per month, and self-report of current daily smoking (yes/no). In addition, physical activity was categorized according to the result obtained in MET-minutes/week using the International Physical Activity Questionnaire (IPAQ). Physical activity levels were categorized in low, medium and high as described previously^14^. Finally, common cardiovascular risk factors were also assessed, including obesity (defined by body mass index ≥30 kg/m^2^), hypertension (systolic blood pressure ≥140 mmHg or diastolic blood pressure ≥90 mmHg, and previous physician diagnosis or currently receiving anti-hypertensive drugs); hypercholesterolemia (total cholesterol level ≥200 mg/dL); and metabolic syndrome^37^.

### Statistical analysis

The statistical package STATA 13 for Windows (StataCorp, College Station, TX, US) was used for analyses. Initially, the description of the study population according to diabetes status at baseline was performed. The comparison showing study population characteristics according to population group is shown in Online Supplement: E-Table 1. Means, standard deviations and proportions of the variables were calculated, and comparisons were performed using the *χ*^2^ test.

For the incidence analysis, we excluded those participants who had been diagnosed with T2DM at baseline. In addition, those lost to follow-up and deaths were also excluded as we could not confirm their T2DM status. In order to calculate incidence rates and associated 95% confidence intervals (95% CI), person-years were calculated by summing the follow-up times for the remaining participants. However, for those participants with a new diagnosis of T2DM, only half of the time between their baseline and follow-up assessment was used in this sum, since the actual date of diagnosis is unknown. We used Poisson regression models, link log function, and robust standard errors to account for cluster effects, reporting risk ratios (RR) and 95% CI. In order to better explore the risk of T2DM in rural-to-urban migrants, we developed two adjusted regression models. Thus, Model 1 includes demographic and urban lifestyle risk factors associated with T2DM as confounders. Model 2 adjusted for the factors in Model 1 and obesity. We explored the effect of obesity in a different model, as obesity increases risks for T2DM development and could be considered as an intermediate variable instead of a confounder^38,39^. In addition, the variance inflation factor was used to evaluate collinearity and exclude variables with high correlation from any of the models, if needed.

### Ethics

The baseline study and subsequent follow-ups were conducted according to the Declaration of Helsinki. An informed consent was obtained from all the participants of the study, detailing the clinical assessment, risk and benefits of participation, confidentiality, and consequent scientific publications. For the rural group, the informed consent was obtained in their mother tongue, Quechua, with local health promoters. The PERU MIGRANT Study baseline study was approved by the Ethics Committees of Universidad Peruana Cayetano Heredia (UPCH) in Peru and the London School of Hygiene and Tropical Medicine in the United Kingdom. In 2015, the Ethics Committee at UPCH approved the follow-up protocol.

## Electronic supplementary material

Supplementary Information

## References

[1] Organization., W. H. WHO | Global status report on noncommunicable diseases 2014Global status report on noncommunicable diseases 2014. (World Health Organization, Geneva. Switzerland, 2015).

[2] GDB 2013 Mortality and Causes of Death Collaborator. Global, regional, and national age-sex specific all-cause and cause-specific mortality for 240 causes of death, 1990–2013: a systematic analysis for the Global Burden of Disease Study 2013. *Lancet* 385, 117–171, 10.1016/s0140-6736(14)61682-2 (2014). 10.1016/S0140-6736(14)61682-2 PMC4340604 25530442

[3] N. C. D. Risk Factor Collaboration. Worldwide trends in diabetes since 1980: a pooled analysis of 751 population-based studies with 4.4 million participants. *Lancet* 387, 1513–1530, 10.1016/s0140-6736(16)00618-8. Epub 2016 Apr 6 (2016). 10.1016/S0140-6736(16)00618-8 PMC5081106 27061677

[4] Pan American Health Organization. Core Indicators 2016. Health Situation in the Americas. (PAHO, Washington DC, US., 2016).

[5] Seclen, S. N., Rosas, M. E., Arias, A. J., Huayta, E. & Medina, C. A. Prevalence of diabetes and impaired fasting glucose in Peru: report from PERUDIAB, a national urban population-based longitudinal study. *BMJ Open Diabetes Res Care 3*, e000110, 10.1136/bmjdrc-2015-000110. eCollection 2015 (2015). 10.1136/bmjdrc-2015-000110 PMC4620143 26512325

[6] Seclen, S. N., Rosas, M. E., Arias, A. J. & Medina, C. A. Elevated incidence rates of diabetes in Peru: report from PERUDIAB, a national urban population-based longitudinal study. *BMJ Open Diabetes Res Care 5*, e000401, 10.1136/bmjdrc-2017-000401. eCollection 2017 (2017). 10.1136/bmjdrc-2017-000401 PMC5574423 28878935

[7] Allender, S., Foster, C., Hutchinson, L. & Arambepola, C. Quantification of urbanization in relation to chronic diseases in developing countries: a systematic review. *J Urban Health* 85, 938–951, 10.1007/s11524-008-9325-4. Epub 2008 Oct 18 (2008). 10.1007/s11524-008-9325-4 PMC2587653 18931915

[8] Mazarello Paes V, Ong KK, Lakshman R (2015). Factors influencing obesogenic dietary intake in young children (0–6 years): systematic review of qualitative evidence. BMJ Open.

[9] Popkin BM, Gordon-Larsen P (2004). The nutrition transition: worldwide obesity dynamics and their determinants. Int J Obes Relat Metab Disord.

[10] Bowen, L. *et al*. Dietary intake and rural-urban migration in India: a cross-sectional study. *PLoS One 6*, e14822, 10.1371/journal.pone.0014822. Epub 2011 Jun 22 (2011). 10.1371/journal.pone.0014822 PMC3120774 21731604

[11] Unwin N (2010). Rural to urban migration and changes in cardiovascular risk factors in Tanzania: a prospective cohort study. BMC Public Health.

[12] Hernandez, A. V., Pasupuleti, V., Deshpande, A., Bernabe-Ortiz, A. & Miranda, J. J. Effect of rural-to-urban within-country migration on cardiovascular risk factors in low- and middle-income countries: a systematic review. Heart 98, 185–194, 10.1136/heartjnl-2011-300599. Epub 2011 Sep 13 (2011). 10.1136/heartjnl-2011-300599 PMC3272377 21917659

[13] Kinra, S. *et al*. Association between urban life-years and cardiometabolic risk: the Indian migration study. *Am J Epidemiol* 174, 154–164, 10.1093/aje/kwr053. Epub 2011 May 27 (2011). 10.1093/aje/kwr053 PMC3132275 21622949

[14] Masterson Creber RM, Smeeth L, Gilman RH, Miranda JJ (2010). Physical activity and cardiovascular risk factors among rural and urban groups and rural-to-urban migrants in Peru: a cross-sectional study. Rev Panam Salud Publica.

[15] Sullivan, R. *et al*. Socio-demographic patterning of physical activity across migrant groups in India: results from the Indian Migration Study. *PLoS One* 6, e24898, 10.1371/journal.pone.0024898. Epub 2011 Oct 14 (2011). 10.1371/journal.pone.0024898 PMC3194815 22022366

[16] Hales CN, Barker DJ (2002). The thrifty phenotype hypothesis. Br Med Bull.

[17] Wells JC (2009). Maternal capital and the metabolic ghetto: An evolutionary perspective on the transgenerational basis of health inequalities. Am J Hum Biol.

[18] Whincup PH (2008). Birth weight and risk of type 2 diabetes: a systematic review. Jama.

[19] Gushulak BD, MacPherson DW (2006). The basic principles of migration health: population mobility and gaps in disease prevalence. Emerg Themes Epidemiol.

[20] Marmot M (1993). Changing places changing risks: the study of migrants. Public Health Rev.

[21] Comisión de la Verdad y Reconciliación. Informe Final de la Comisión de la Verdad y Reconciliación. (Comisión de la Verdad y Reconciliación., Lima, Peru, 2003).

[22] Coral, I. Desplazamiento por violencia política en el Perú, 1980–1992. (INSTITUTO DE ESTUDIOS PERUANOS, Lima, Peru, 1994).

[23] Miranda JJ, Gilman RH, Garcia HH, Smeeth L (2009). The effect on cardiovascular risk factors of migration from rural to urban areas in Peru: PERU MIGRANT Study. BMC Cardiovasc Disord.

[24] Guo, S., Lucas, R. M., Joshy, G. & Banks, E. Cardiovascular disease risk factor profiles of 263,356 older Australians according to region of birth and acculturation, with a focus on migrants born in Asia. *PLoS One* 10, e0115627, 10.1371/journal.pone.0115627. eCollection 2015 (2015). 10.1371/journal.pone.0115627 PMC4335012 25695771

[25] Zheng, Y. *et al*. Impact of migration and acculturation on prevalence of type 2 diabetes and related eye complications in Indians living in a newly urbanised society. *PLoS One* 7, e34829, 10.1371/journal.pone.0034829. Epub 2012 Apr 10 (2012). 10.1371/journal.pone.0034829 PMC3323593 22506053

[26] Commodore-Mensah, Y. *et al*. Length of Residence in the United States is Associated With a Higher Prevalence of Cardiometabolic Risk Factors in Immigrants: A Contemporary Analysis of the National Health Interview Survey. *J Am Heart Assoc* 5, 10.1161/jaha.116.004059 (2016). 10.1161/JAHA.116.004059 PMC5210341 27815269

[27] Jaffe, A. *et al*. Diabetes among Ethiopian Immigrants to Israel: Exploring the Effects of Migration and Ethnicity on Diabetes Risk. *PLoS One* 11, e0157354, 10.1371/journal.pone.0157354. eCollection 2016 (2016). 10.1371/journal.pone.0157354 PMC4907509 27300299

[28] Azimi-Nezhad M (2008). Prevalence of type 2 diabetes mellitus in Iran and its relationship with gender, urbanisation, education, marital status and occupation. Singapore Med J.

[29] Ebrahim S (2010). The effect of rural-to-urban migration on obesity and diabetes in India: a cross-sectional study. PLoS Med.

[30] Bernabe-Ortiz, A. *et al*. Geographical variation in the progression of type 2 diabetes in Peru: The CRONICAS Cohort Study. *Diabetes Res Clin Pract* 121, 135–145, 10.1016/j.diabres.2016.09.007. Epub 2016 Sep 21 (2016). 10.1016/j.diabres.2016.09.007 PMC5154928 27710820

[31] Carrillo-Larco, R. M. *et al*. Obesity risk in rural, urban and rural-to-urban migrants: prospective results of the PERU MIGRANT study. Int J Obes (Lond) 40, 181–185, 10.1038/ijo.2015.140. Epub2015 Jul 31 (2015). 10.1038/ijo.2015.140 PMC4677453 26228458

[32] Townshend, T. & Lake, A. A. Obesogenic urban form: theory, policy and practice. *Health Place* 15, 909–916, 10.1016/j.healthplace.2008.12.002. Epub 2008 Dec 25 (2009). 10.1016/j.healthplace.2008.12.002 19201641

[33] Mackenbach JD (2014). Obesogenic environments: a systematic review of the association between the physical environment and adult weight status, the SPOTLIGHT project. BMC Public Health.

[34] Fennelly K (2007). The “healthy migrant” effect. Minn Med.

[35] Antiporta, D. A., Smeeth, L., Gilman, R. H. & Miranda, J. J. Length of urban residence and obesity among within-country rural-to-urban Andean migrants. *Public Health Nutr* 19, 1270–1278, 10.1017/s1368980015002578. Epub 2015 Sep 14 (2015). 10.1017/S1368980015002578 PMC4825096 26365215

[36] Carrillo-Larco RM (2017). Cohort Profile: The PERU MIGRANT Study-A prospective cohort study of rural dwellers, urban dwellers and rural-to-urban migrants in Peru. Int J Epidemiol.

[37] Alberti, K. G. *et al*. Harmonizing the metabolic syndrome: a joint interim statement of the International Diabetes Federation Task Force on Epidemiology and Prevention; National Heart, Lung, and Blood Institute; American Heart Association; World Heart Federation; International Atherosclerosis Society; and International Association for the Study of Obesity. *Circulation* 120, 1640–1645, 10.1161/circulationaha.109.192644. Epub 2009 Oct 5 (2009). 10.1161/CIRCULATIONAHA.109.192644 19805654

[38] Wild SH, Byrne CD (2006). ABC of obesity. Risk factors for diabetes and coronary heart disease. Bmj.

[39] VanderWeele TJ, Hernan MA, Robins JM (2008). Causal directed acyclic graphs and the direction of unmeasured confounding bias. Epidemiology.

