# Supplementary material for: Addressing the impact of urban exposure on the incidence of type 2 diabetes mellitus: The PERU MIGRANT Study
Source: Sci Rep. 2018 Apr 3;8:5512. doi: 10.1038/s41598-018-23812-6 (PMC5883030; doi:10.1038/s41598-018-23812-6)
Supplement: Supplementary file 1 — Supplementary Information [file 41598_2018_23812_MOESM1_ESM.doc]

# **Addressing the impact of urban exposure on the incidence of type 2 diabetes mellitus: The PERU MIGRANT Study**

Andrea Ruiz-Alejos, MD^1^; Rodrigo M. Carrillo-Larco, MD^1^; J. Jaime Miranda, PhD^1,2^; Cheryl A.M. Anderson, PhD^3^; Robert H. Gilman, MD^1,4^; Liam Smeeth, PhD^5^; Antonio Bernabé-Ortiz, MPH^1,5^

**E-Table 1:** Study population characteristics by population group

|  | **PERU MIGRANT study groups** | | |  |
| --- | --- | --- | --- | --- |
|  | **Rural** | **Migrant** | **Urban** | **p-value** |
| ***Sex, n (%)*** | N=201 | N=589 | N=199 |  |
| Female | 106 (52.7) | 309 (52.5) | 107 (53.8) | 0.95 |
| ***Age, n (%)*** | N=201 | N=589 | N=199 |  |
| <50 | 117 (58.2) | 337 (57.2) | 110 (55.3) | 0.83 |
| +50 | 84 (41.8) | 252 (42.8) | 89 (44.7) |  |
| ***Asset index, n (%)*** | N=201 | N=589 | N=199 |  |
| Low | 124 (61.7) | 242 (41.1) | 67 (33.7) | <0.001 |
| Middle | 14 (7.0) | 156 (26.5) | 69 (34.7) |  |
| High | 63 (31.3) | 191 (32.4) | 63 (31.7) |  |
| ***Education, n (%)*** | N=201 | N=588 | N=198 |  |
| None/Some primary | 132 (65.7) | 183 (31.1) | 13 (6.6) | <0.001 |
| Primary complete | 30 (14.9) | 99 (16.8) | 23 (11.6) |  |
| Some secondary or more | 39 (19.4) | 306 (52.0) | 162 (81.8) |  |
| ***Current daily smoking, n (%)*** | N=201 | N=587 | N=199 |  |
| Yes | 1 (0.5) | 15 (2.6) | 17 (8.5) | <0.001 |
| ***Heavy alcohol drinking, n (%)*** | N=201 | N=589 | N=199 |  |
| Yes | 25 (12.4) | 48 (8.2) | 19 (9.6) | 0.19 |
| ***Physical activity levels, n (%)*** | N=201 | N=582 | N=198 |  |
| Low | 4 (2.0) | 173 (29.7) | 78 (39.4) | <0.001 |
| ***Obesity, n (%)*** | N=201 | N=589 | N=199 |  |
| Yes | 6 (3.0) | 124 (21.1) | 68 (34.2) | <0.001 |
| ***Metabolic syndrome, n (%)*** | N=201 | N=589 | N=199 |  |
| Yes | 23 (11.4) | 193 (32.8) | 89 (44.7) | <0.001 |
| ***Hypertension, n (%)*** | N=201 | N=588 | N=199 |  |
| Yes | 24 (11.9) | 76 (12.9) | 59 (29.7) | <0.001 |
| ***Hypercholesterolemia, n (%)*** | N=201 | N=588 | N=199 |  |
| Yes | 15 (7.5) | 220 (37.4) | 71 (35.7) | <0.001 |
| ***Type 2 diabetes, n (%)*** | N=200 | N=589 | N=199 |  |
| Yes | 3 (1.5) | 21 (3.6) | 16 (8.0) | 0.003 |

Results might not add due to missing values.
